# Editorial: Injectable and biodegradable nanocomposite hydrogels: Outlooks and opportunities

**DOI:** 10.3389/fbioe.2022.1096391

**Published:** 2022-12-01

**Authors:** Ling Wang, Meng Yu, Yajuan Su

**Affiliations:** ^1^ Biomaterials Research Center, School of Biomedical Engineering, Southern Medical University, Guangzhou, China; ^2^ State Key Laboratory for Mechanical Behavior of Materials, Xi’an Jiaotong University, Xi’an, China; ^3^ Department of Surgery-Transplant and Holland Regenerative Medicine Program, University of Nebraska Medical Center, Omaha, NE, United States

**Keywords:** injectable hydrogel, nanocomposite hydrogel, tissue engineering, wound healing, drug delivery

Injectable and biodegradable hydrogels have been regarded as encouraging materials in the biomedical field because they had a similar three-dimensional microenvironment with native tissues and cells that could be effortlessly encapsulated within these hydrogels dispersedly. During the period of tissue regeneration, biodegradable hydrogels could be degraded with the deposition of extracellular matrix secreted by cells, thus serving as a promising “temporary extracellular matrix” in remodeling. However, due to the lack of precise microarchitecture control and the limited ability to reconstruct living structures containing complex anisotropic microenvironment, hydrogels with precise topographic cues remains an ongoing challenge. Considering these shortcomings, nanocomposite hydrogels which consisted of nanomaterials and hydrogels enable improve directed structure formation at the multiscale. The integration of nanomaterials, such as nanofibers, nanoparticles, nanoclay and nanogel, and hydrogel endow new mechanical and functional properties, which will be highly beneficial for various biomedical applications, especially tissue engineering and regeneration.

Here, this Research Topic collected a total of four original research papers, which described different kinds of injectable hydrogels from the design, preparation and evaluation. The papers on this Research Topic are briefly shared below.

Silk fibroin (SF) materials have been applied as an appealing biomaterial in the biomedical and pharmaceutical area over the past few decades. Haghighattalab et al. added magnetic nanoparticles (MNPs) into silk fibroin hydrogel and loaded with doxorubicin hydrochloride (DOX) in the meantime using a straightforward blending method to determine the capacity of controlled drug release under the diversified external magnetic field (EMF). With a higher DOX concentration, the Wilms’ tumor cells showed drug resistance which revealed the importance of controlled drug delivery. This study demonstrated that the magnetic SF hydrogel provided a new strategy for smart drug delivery with non-invasive injection and remote control capability.

It is urgent to develop a potent coagulation and anti-infection strategy save patients suffering from wounds or trauma. Hydrogels are distinguished as the most suitable biomaterials for accelerating wound healing. Wang et al. designed an injectable hydrogel based on Bionic Self-Assembling Peptide (BSAP) with hemostatic and bactericidal function for wound healing. BSAP could co-assemble swiftly into a hydrogel network structure driven by Ca^2+^ *in situ*. This hydrogel displayed a lower blood clotting index and reduced blood clotting time and bleeding volume dramatically in the foot trauma model and tail amputation model. Besides, by fighting bacterial infection, this hydrogel could reduce inflammatory responses and enhance wound healing.

To achieve better mechanical match and stronger biological properties of bone biomaterials, Wang et al. prepared a new organic-inorganic composite hydrogel (FPIGP@BGN-Sr hydrogel) comprising diacrylated PF-127, β-GP-modified polyitaconate and BGN-Sr *via* radical polymerization and coordination interactions. The composite hydrogel could effectively enhance the mouse embryonic mesenchymal stem cells’ osteogenic differentiation Therefore, the appropriate concentration of BGN-Sr, PF-127 and β-GP-modified polyitaconate exhibited good synergistic effects on promoting osteogenic differentiation of MSCs, therefore offered a promising approach for stem cell-based bone regeneration.

Chen et al. fabricated an injectable mesh using N-isopropylacrylamide (NIPAAm), N-hydroxyethyl acrylamide (HMAAm) and Polyvinyl alcohol (PVA) based on the electrospinning method. The temperature-responsive mesh could be injected at the tumor site to modulate and control anticancer effects by loading magnetic nanoparticles (MNPs) and chemotherapeutic drugs such as paclitaxel (PTX). This study revealed a new therapeutic platform for cancer treatment using hyperthermia combined with chemotherapy.

Many advances have been made in the application of injectable and biodegradable hydrogels in the treatment of various diseases, tissue regeneration and controlled release of drugs. However, the application of injectable biodegradable hydrogels was still restricted by some practical problems. For example, the needle loaded with temperature-responsive hydrogels may be blocked when injected subcutaneously due to the temperature increase, thus causing a failed injection. Moreover, the long-term storage and stability of injectable and biodegradable hydrogels still needed to be improved. To solve these dilemmas, developments of injectable and biodegradable hydrogels that respond to other stimuli, such as hypoxia, light, or enzymes, may be alternative strategies. Most current studies focus on improving the performance of biodegradable and injectable hydrogels. However, the degradation of products’ toxicity is an overlooked problem. Therefore, more work needs to be done to evaluate the safety of biodegradable hydrogels after the practical application tasks are completed. Future work should also focus on the fabrication of biodegradable and injectable hydrogels using more convenient, cost-effective, and environmentally friendly methods. Additionally, the development of biodegradable and injectable hydrogels with more advanced properties should be further explored to provide more options in various application scenarios. Through these efforts, biodegradable and injectable hydrogels will benefit an increasing number of patients in the near future.

